# Di-*tert*-butyl 3,5-dimethyl-1*H*-pyrrole-2,4-dicarboxyl­ate

**DOI:** 10.1107/S1600536812026700

**Published:** 2012-06-20

**Authors:** Zhao-Po Zhang, Wei-Na Wu

**Affiliations:** a Department of Physics and Chemistry, Henan Polytechnic University, Jiaozuo 454000, People’s Republic of China

## Abstract

In the title mol­ecule, C_16_H_25_NO_4_, the non-H atoms, except for the two *tert*-butyl groups, are roughly planar (r.m.s. deviation of the non-H atoms = 0.086 Å). In the crystal, mol­ecules are linked into inversion dimers by pairs of N—H⋯O hydrogen bonds, forming *R*_2_^2^(10) ring motifs.

## Related literature

For complexes of Schiff bases containing a pyrrole unit, see: Wu *et al.* (2003 ▶); Wang *et al.* (2008 ▶). For the synthesis of the title compound, see: Sun *et al.* (2003 ▶).
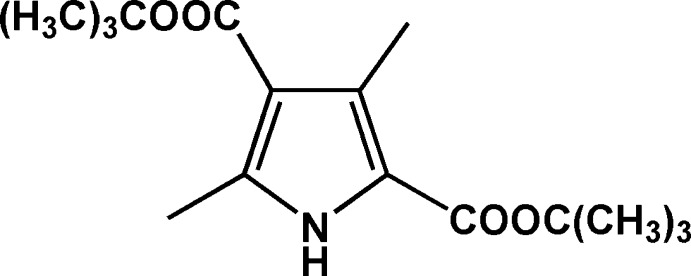


## Experimental

### 

#### Crystal data

C_16_H_25_NO_4_
*M* *_r_* = 295.37
Triclinic, 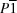

*a* = 5.8976 (10) Å
*b* = 11.511 (2) Å
*c* = 13.460 (2) Å
α = 103.956 (4)°
β = 90.078 (3)°
γ = 104.804 (3)°
*V* = 855.5 (3) Å^3^
*Z* = 2
Mo *K*α radiation
μ = 0.08 mm^−1^
*T* = 296 K
0.21 × 0.19 × 0.16 mm

#### Data collection

Bruker SMART CCD diffractometer
Absorption correction: multi-scan (*SADABS*; Bruker, 2007 ▶) *T*_min_ = 0.983, *T*_max_ = 0.987
4496 measured reflections
2989 independent reflections
1704 reflections with *I* > 2σ(*I*)
*R*_int_ = 0.023

#### Refinement

*R*[*F* ^2^ > 2σ(*F* ^2^)] = 0.058
*wR*(*F* ^2^) = 0.150
*S* = 1.04
2989 reflections
191 parameters
H atoms treated by a mixture of independent and constrained refinement
Δρ_max_ = 0.27 e Å^−3^
Δρ_min_ = −0.21 e Å^−3^

### 

Data collection: *APEX2* (Bruker, 2007 ▶); cell refinement: *SAINT* (Bruker, 2007 ▶); data reduction: *SAINT*; program(s) used to solve structure: *SHELXS97* (Sheldrick, 2008 ▶); program(s) used to refine structure: *SHELXL97* (Sheldrick, 2008 ▶); molecular graphics: *SHELXTL* (Sheldrick, 2008 ▶); software used to prepare material for publication: *SHELXTL*.

## Supplementary Material

Crystal structure: contains datablock(s) I, global. DOI: 10.1107/S1600536812026700/vm2177sup1.cif

Structure factors: contains datablock(s) I. DOI: 10.1107/S1600536812026700/vm2177Isup2.hkl

Supplementary material file. DOI: 10.1107/S1600536812026700/vm2177Isup3.cml

Additional supplementary materials:  crystallographic information; 3D view; checkCIF report

## Figures and Tables

**Table 1:** Hydrogen-bond geometry (Å, °)

*D*—H⋯*A*	*D*—H	H⋯*A*	*D*⋯*A*	*D*—H⋯*A*
N1—H1*A*⋯O1^i^	0.873 (17)	2.087 (18)	2.933 (3)	163.2 (12)

Symmetry code: (i) 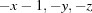.

**Fig. 1.**
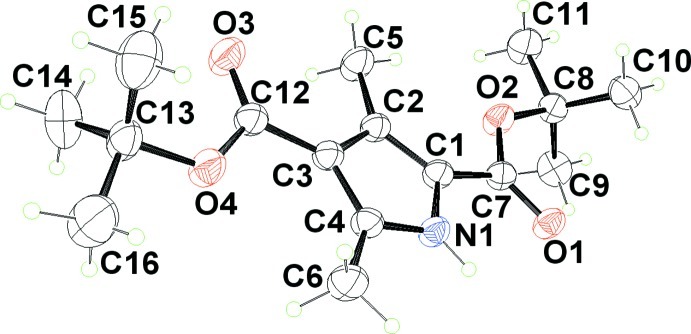
The molecular structure shown with 30% probability displacement ellipsoids.

**Fig. 2.**
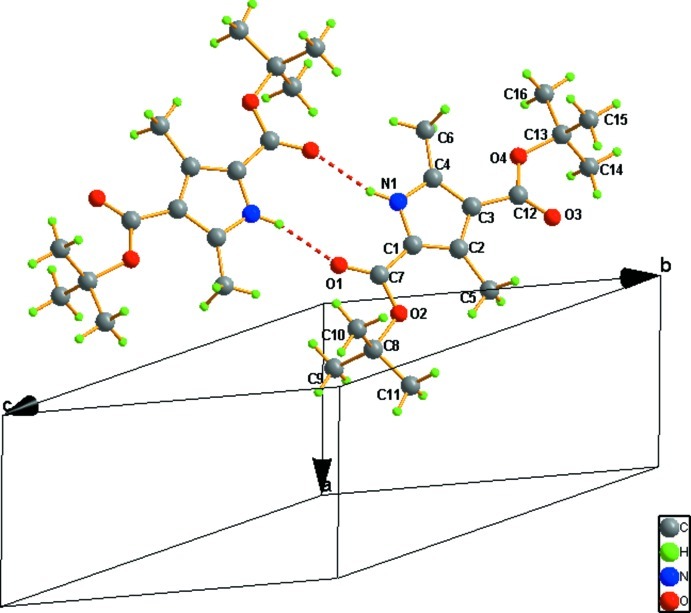
The dimer of the title compounds formed via N—H···O hydrogen bonds shown as dashed lines. Unlabelled atoms are related with the labelled ones by symmetry operation -1 - x, -y, -z.

## supplementary crystallographic information

### Comment

Schiff bases containing pyrrole units have been extensively investigated due to their excellent coordination abilities (Wu *et al.*, 2003; Wang *et al.*, 2008). However, *tert*-butyl pyrrole-2-carboxylate derivatives are important intermediates to form 2-formyl pyrroles (Sun *et al.*, 2003). As part of our studies on bis(pyrrol-2-yl-methyleneamine) ligands, the crystal structure of the title compound is reported here.

In the title molecule (Fig. 1), except for the two *tert*-butyl groups, the non-hydrogen atoms are situated in a fair plane (r.m.s. deviation of the non-hydrogen atoms being 0.2542 Å). In the crystal, the molecules are linked into a centrosymmetric dimer by two intermolecular N—H···O hydrogen bonds (Table 1), forming a *R*_2_^2^(10) ring motif (Fig. 2).

### Experimental

The di-*tert*-butyl 3,5-dimethyl-1*H*-pyrrole-2,4-dicarboxylate was prepared by a Knorr-type reaction from the condensation of *tert*-butyl acetoacetate and *tert*-butyl oximinoacetoacetate according to literature (Sun *et al.*, 2003).

### Refinement

All methyl H atoms were positioned geometrically (C—H = 0.96 Å) and refined as riding with *U*_iso_(H) = 1.5U_eq_. Atom H1A was positioned geometrically with the N1—H1A distance free to refine and U_iso_(H1A) = 1.2U_eq_(N1).

### Crystal data

**Table:**

C_16_H_25_NO_4_	*Z* = 2
*M**_r_* = 295.37	*F*(000) = 320
Triclinic, *P*1	*D*_x_ = 1.147 Mg m^−^^3^
Hall symbol: -P 1	Mo *K*α radiation, λ = 0.71073 Å
*a* = 5.8976 (10) Å	Cell parameters from 771 reflections
*b* = 11.511 (2) Å	θ = 3.2–20.5°
*c* = 13.460 (2) Å	µ = 0.08 mm^−^^1^
α = 103.956 (4)°	*T* = 296 K
β = 90.078 (3)°	Plate, colorless
γ = 104.804 (3)°	0.21 × 0.19 × 0.16 mm
*V* = 855.5 (3) Å^3^	

### Data collection

**Table:**

Bruker SMART CCD diffractometer	2989 independent reflections
Radiation source: fine-focus sealed tube	1704 reflections with *I* > 2σ(*I*)
Graphite monochromator	*R*_int_ = 0.023
φ and ω scans	θ_max_ = 25.0°, θ_min_ = 1.9°
Absorption correction: multi-scan (*SADABS*; Bruker, 2007)	*h* = −6→6
*T*_min_ = 0.983, *T*_max_ = 0.987	*k* = −13→13
4496 measured reflections	*l* = −16→13

### Refinement

**Table:**

Refinement on *F*^2^	Primary atom site location: structure-invariant direct methods
Least-squares matrix: full	Secondary atom site location: difference Fourier map
*R*[*F*^2^ > 2σ(*F*^2^)] = 0.058	Hydrogen site location: inferred from neighbouring sites
*wR*(*F*^2^) = 0.150	H atoms treated by a mixture of independent and constrained refinement
*S* = 1.04	*w* = 1/[σ^2^(*F*_o_^2^) + (0.0557*P*)^2^ + 0.202*P*] where *P* = (*F*_o_^2^ + 2*F*_c_^2^)/3
2989 reflections	(Δ/σ)_max_ < 0.001
191 parameters	Δρ_max_ = 0.27 e Å^−^^3^
0 restraints	Δρ_min_ = −0.21 e Å^−^^3^

### Special details

**Table:**

Geometry. All e.s.d.'s (except the e.s.d. in the dihedral angle between two l.s. planes) are estimated using the full covariance matrix. The cell e.s.d.'s are taken into account individually in the estimation of e.s.d.'s in distances, angles and torsion angles; correlations between e.s.d.'s in cell parameters are only used when they are defined by crystal symmetry. An approximate (isotropic) treatment of cell e.s.d.'s is used for estimating e.s.d.'s involving l.s. planes.
Refinement. Refinement of *F*^2^ against ALL reflections. The weighted *R*-factor *wR* and goodness of fit *S* are based on *F*^2^, conventional *R*-factors *R* are based on *F*, with *F* set to zero for negative *F*^2^. The threshold expression of *F*^2^ > σ(*F*^2^) is used only for calculating *R*-factors(gt) *etc*. and is not relevant to the choice of reflections for refinement. *R*-factors based on *F*^2^ are statistically about twice as large as those based on *F*, and *R*- factors based on ALL data will be even larger.

### Fractional atomic coordinates and isotropic or equivalent isotropic displacement parameters (Å^2^)

**Table:**

	*x*	*y*	*z*	*U*_iso_*/*U*_eq_	
C1	−0.2311 (4)	0.1845 (2)	−0.08222 (19)	0.0471 (7)	
C2	−0.1610 (4)	0.2579 (2)	−0.14921 (19)	0.0476 (7)	
C3	−0.3401 (5)	0.2180 (2)	−0.23018 (19)	0.0478 (7)	
C4	−0.5147 (5)	0.1215 (2)	−0.2090 (2)	0.0484 (7)	
C5	0.0637 (5)	0.3586 (3)	−0.1403 (2)	0.0673 (9)	
H5A	0.1527	0.3670	−0.0781	0.101*	
H5B	0.0276	0.4356	−0.1393	0.101*	
H5C	0.1540	0.3375	−0.1980	0.101*	
C6	−0.7463 (5)	0.0472 (3)	−0.2626 (2)	0.0691 (9)	
H6A	−0.8212	−0.0121	−0.2255	0.104*	
H6B	−0.7229	0.0045	−0.3308	0.104*	
H6C	−0.8440	0.1015	−0.2660	0.104*	
C7	−0.1316 (4)	0.1787 (2)	0.0145 (2)	0.0479 (7)	
C8	0.1962 (5)	0.2870 (3)	0.1451 (2)	0.0537 (7)	
C9	0.2769 (5)	0.1727 (3)	0.1469 (2)	0.0726 (9)	
H9A	0.3721	0.1553	0.0903	0.109*	
H9B	0.1424	0.1032	0.1415	0.109*	
H9C	0.3674	0.1870	0.2101	0.109*	
C10	0.0441 (5)	0.3201 (3)	0.2315 (2)	0.0693 (9)	
H10A	−0.0005	0.3935	0.2274	0.104*	
H10B	0.1301	0.3351	0.2960	0.104*	
H10C	−0.0944	0.2529	0.2263	0.104*	
C11	0.4029 (6)	0.3960 (3)	0.1429 (3)	0.0819 (10)	
H11A	0.3469	0.4670	0.1417	0.123*	
H11B	0.4861	0.3754	0.0827	0.123*	
H11C	0.5067	0.4147	0.2030	0.123*	
C12	−0.3408 (5)	0.2728 (3)	−0.3174 (2)	0.0549 (7)	
C13	−0.5671 (6)	0.2464 (3)	−0.4787 (2)	0.0673 (9)	
C14	−0.3574 (7)	0.2685 (4)	−0.5428 (3)	0.1043 (13)	
H14A	−0.3103	0.1930	−0.5657	0.156*	
H14B	−0.2298	0.3317	−0.5022	0.156*	
H14C	−0.3990	0.2948	−0.6012	0.156*	
C15	−0.6498 (7)	0.3616 (4)	−0.4426 (3)	0.1023 (13)	
H15A	−0.7827	0.3448	−0.4023	0.153*	
H15B	−0.6943	0.3873	−0.5009	0.153*	
H15C	−0.5252	0.4265	−0.4017	0.153*	
C16	−0.7630 (8)	0.1391 (4)	−0.5360 (3)	0.1329 (19)	
H16A	−0.8932	0.1267	−0.4935	0.199*	
H16B	−0.7085	0.0654	−0.5536	0.199*	
H16C	−0.8123	0.1567	−0.5976	0.199*	
N1	−0.4459 (4)	0.10303 (19)	−0.12068 (16)	0.0492 (6)	
H1A	−0.526 (2)	0.0471 (17)	−0.0913 (9)	0.059*	
O1	−0.2209 (3)	0.10146 (17)	0.06093 (14)	0.0565 (5)	
O2	0.0654 (3)	0.26788 (16)	0.04613 (13)	0.0594 (6)	
O3	−0.2064 (4)	0.3672 (2)	−0.32547 (15)	0.0789 (7)	
O4	−0.5120 (4)	0.20430 (18)	−0.38930 (14)	0.0728 (7)	

### Atomic displacement parameters (Å^2^)

**Table:**

	*U*^11^	*U*^22^	*U*^33^	*U*^12^	*U*^13^	*U*^23^
C1	0.0392 (15)	0.0471 (16)	0.0494 (16)	0.0026 (13)	−0.0034 (13)	0.0109 (13)
C2	0.0431 (16)	0.0459 (16)	0.0501 (16)	0.0040 (13)	0.0011 (13)	0.0133 (13)
C3	0.0487 (17)	0.0433 (15)	0.0484 (16)	0.0059 (13)	−0.0017 (13)	0.0123 (12)
C4	0.0481 (17)	0.0447 (16)	0.0490 (16)	0.0062 (13)	−0.0060 (13)	0.0117 (13)
C5	0.0580 (19)	0.069 (2)	0.066 (2)	−0.0086 (16)	−0.0064 (16)	0.0271 (16)
C6	0.062 (2)	0.065 (2)	0.068 (2)	−0.0084 (16)	−0.0152 (16)	0.0191 (16)
C7	0.0364 (15)	0.0501 (16)	0.0511 (16)	0.0022 (13)	−0.0015 (13)	0.0109 (14)
C8	0.0435 (16)	0.0598 (18)	0.0503 (17)	0.0019 (14)	−0.0090 (13)	0.0121 (14)
C9	0.056 (2)	0.088 (2)	0.078 (2)	0.0266 (18)	−0.0060 (16)	0.0190 (18)
C10	0.068 (2)	0.070 (2)	0.064 (2)	0.0160 (17)	0.0038 (17)	0.0075 (16)
C11	0.058 (2)	0.089 (2)	0.079 (2)	−0.0151 (18)	−0.0104 (17)	0.0205 (18)
C12	0.0551 (18)	0.0514 (18)	0.0541 (17)	0.0048 (15)	−0.0033 (15)	0.0152 (14)
C13	0.067 (2)	0.075 (2)	0.0572 (19)	0.0036 (17)	−0.0113 (17)	0.0290 (16)
C14	0.103 (3)	0.154 (4)	0.063 (2)	0.040 (3)	0.009 (2)	0.033 (2)
C15	0.105 (3)	0.133 (4)	0.092 (3)	0.050 (3)	0.004 (2)	0.050 (2)
C16	0.137 (4)	0.125 (3)	0.107 (3)	−0.041 (3)	−0.073 (3)	0.053 (3)
N1	0.0457 (14)	0.0490 (13)	0.0491 (13)	0.0005 (11)	−0.0029 (11)	0.0181 (11)
O1	0.0495 (12)	0.0582 (12)	0.0572 (12)	−0.0030 (9)	−0.0060 (9)	0.0240 (10)
O2	0.0486 (12)	0.0636 (13)	0.0566 (12)	−0.0083 (10)	−0.0105 (9)	0.0220 (9)
O3	0.0853 (16)	0.0726 (15)	0.0676 (14)	−0.0133 (13)	−0.0132 (12)	0.0327 (11)
O4	0.0795 (15)	0.0684 (14)	0.0620 (13)	−0.0075 (11)	−0.0240 (11)	0.0292 (11)

### Geometric parameters (Å, º)

**Table:**

C1—C2	1.375 (3)	C9—H9C	0.9600
C1—N1	1.381 (3)	C10—H10A	0.9600
C1—C7	1.450 (4)	C10—H10B	0.9600
C2—C3	1.422 (3)	C10—H10C	0.9600
C2—C5	1.504 (3)	C11—H11A	0.9600
C3—C4	1.394 (3)	C11—H11B	0.9600
C3—C12	1.462 (4)	C11—H11C	0.9600
C4—N1	1.337 (3)	C12—O3	1.200 (3)
C4—C6	1.490 (3)	C12—O4	1.342 (3)
C5—H5A	0.9600	C13—O4	1.466 (3)
C5—H5B	0.9600	C13—C15	1.502 (5)
C5—H5C	0.9600	C13—C16	1.505 (4)
C6—H6A	0.9600	C13—C14	1.512 (5)
C6—H6B	0.9600	C14—H14A	0.9600
C6—H6C	0.9600	C14—H14B	0.9600
C7—O1	1.220 (3)	C14—H14C	0.9600
C7—O2	1.328 (3)	C15—H15A	0.9600
C8—O2	1.479 (3)	C15—H15B	0.9600
C8—C10	1.506 (4)	C15—H15C	0.9600
C8—C9	1.514 (4)	C16—H16A	0.9600
C8—C11	1.516 (4)	C16—H16B	0.9600
C9—H9A	0.9600	C16—H16C	0.9600
C9—H9B	0.9600	N1—H1A	0.873 (17)
			
C2—C1—N1	107.7 (2)	C8—C10—H10C	109.5
C2—C1—C7	134.1 (2)	H10A—C10—H10C	109.5
N1—C1—C7	118.2 (2)	H10B—C10—H10C	109.5
C1—C2—C3	106.5 (2)	C8—C11—H11A	109.5
C1—C2—C5	126.9 (2)	C8—C11—H11B	109.5
C3—C2—C5	126.6 (2)	H11A—C11—H11B	109.5
C4—C3—C2	107.8 (2)	C8—C11—H11C	109.5
C4—C3—C12	127.0 (2)	H11A—C11—H11C	109.5
C2—C3—C12	125.2 (2)	H11B—C11—H11C	109.5
N1—C4—C3	107.2 (2)	O3—C12—O4	123.1 (3)
N1—C4—C6	120.3 (2)	O3—C12—C3	125.1 (3)
C3—C4—C6	132.5 (2)	O4—C12—C3	111.8 (2)
C2—C5—H5A	109.5	O4—C13—C15	108.9 (3)
C2—C5—H5B	109.5	O4—C13—C16	102.4 (2)
H5A—C5—H5B	109.5	C15—C13—C16	111.3 (3)
C2—C5—H5C	109.5	O4—C13—C14	111.3 (3)
H5A—C5—H5C	109.5	C15—C13—C14	111.4 (3)
H5B—C5—H5C	109.5	C16—C13—C14	111.2 (3)
C4—C6—H6A	109.5	C13—C14—H14A	109.5
C4—C6—H6B	109.5	C13—C14—H14B	109.5
H6A—C6—H6B	109.5	H14A—C14—H14B	109.5
C4—C6—H6C	109.5	C13—C14—H14C	109.5
H6A—C6—H6C	109.5	H14A—C14—H14C	109.5
H6B—C6—H6C	109.5	H14B—C14—H14C	109.5
O1—C7—O2	124.6 (2)	C13—C15—H15A	109.5
O1—C7—C1	123.5 (2)	C13—C15—H15B	109.5
O2—C7—C1	111.9 (2)	H15A—C15—H15B	109.5
O2—C8—C10	109.2 (2)	C13—C15—H15C	109.5
O2—C8—C9	109.9 (2)	H15A—C15—H15C	109.5
C10—C8—C9	112.8 (2)	H15B—C15—H15C	109.5
O2—C8—C11	101.8 (2)	C13—C16—H16A	109.5
C10—C8—C11	111.4 (2)	C13—C16—H16B	109.5
C9—C8—C11	111.2 (3)	H16A—C16—H16B	109.5
C8—C9—H9A	109.5	C13—C16—H16C	109.5
C8—C9—H9B	109.5	H16A—C16—H16C	109.5
H9A—C9—H9B	109.5	H16B—C16—H16C	109.5
C8—C9—H9C	109.5	C4—N1—C1	110.9 (2)
H9A—C9—H9C	109.5	C4—N1—H1A	124.5 (8)
H9B—C9—H9C	109.5	C1—N1—H1A	124.6 (8)
C8—C10—H10A	109.5	C7—O2—C8	122.9 (2)
C8—C10—H10B	109.5	C12—O4—C13	122.3 (2)
H10A—C10—H10B	109.5		
			
N1—C1—C2—C3	−0.3 (3)	C2—C3—C12—O3	−9.7 (5)
C7—C1—C2—C3	−179.0 (3)	C4—C3—C12—O4	−12.0 (4)
N1—C1—C2—C5	−178.9 (2)	C2—C3—C12—O4	170.3 (2)
C7—C1—C2—C5	2.5 (5)	C3—C4—N1—C1	0.2 (3)
C1—C2—C3—C4	0.4 (3)	C6—C4—N1—C1	−177.7 (2)
C5—C2—C3—C4	179.0 (3)	C2—C1—N1—C4	0.1 (3)
C1—C2—C3—C12	178.5 (3)	C7—C1—N1—C4	179.0 (2)
C5—C2—C3—C12	−3.0 (4)	O1—C7—O2—C8	−2.5 (4)
C2—C3—C4—N1	−0.3 (3)	C1—C7—O2—C8	176.8 (2)
C12—C3—C4—N1	−178.3 (3)	C10—C8—O2—C7	−63.0 (3)
C2—C3—C4—C6	177.1 (3)	C9—C8—O2—C7	61.2 (3)
C12—C3—C4—C6	−0.9 (5)	C11—C8—O2—C7	179.2 (2)
C2—C1—C7—O1	−177.3 (3)	O3—C12—O4—C13	−7.2 (5)
N1—C1—C7—O1	4.1 (4)	C3—C12—O4—C13	172.8 (3)
C2—C1—C7—O2	3.3 (4)	C15—C13—O4—C12	−63.2 (4)
N1—C1—C7—O2	−175.2 (2)	C16—C13—O4—C12	178.9 (3)
C4—C3—C12—O3	168.0 (3)	C14—C13—O4—C12	60.0 (4)

### Hydrogen-bond geometry (Å, º)

**Table:**

*D*—H···*A*	*D*—H	H···*A*	*D*···*A*	*D*—H···*A*
N1—H1*A*···O1^i^	0.873 (17)	2.087 (18)	2.933 (3)	163.2 (12)

Symmetry code: (i) −*x*−1, −*y*, −*z*.
